# Temperature and Pressure Dependence of Gas Permeation in a Microporous Tröger’s Base Polymer

**DOI:** 10.3390/membranes8040132

**Published:** 2018-12-14

**Authors:** Elsa Lasseuguette, Richard Malpass-Evans, Mariolino Carta, Neil B. McKeown, Maria-Chiara Ferrari

**Affiliations:** 1 School of Engineering, University of Edinburgh, Robert Stevenson Road, Edinburgh EH9 3FB, UK; e.lasseuguette@ed.ac.uk; 2 EastChem, School of Chemistry, University of Edinburgh, David Brewster Road, Edinburgh EH9 3FJ, UK; R.Malpass-Evans@ed.ac.uk (R.M.-E.); Neil.McKeown@ed.ac.uk (N.B.M.); 3 Department of Chemistry, College of Science, Grove Building, Singleton Park, Swansea University, Swansea SA2 8PP, UK; mariolino.carta@swansea.ac.uk

**Keywords:** microporous polymer, gas permeability, activation energy, CO_2_ capture

## Abstract

Gas transport properties of PIM-EA(H_2_)-TB, a microporous Tröger’s base polymer, were systematically studied over a range of pressure and temperature. Permeability coefficients of pure CO_2_, N_2_, CH_4_ and H_2_ were determined for upstream pressures up to 20 bar and temperatures up to 200 °C. PIM-EA(H_2_)-TB exhibited high permeability coefficients in absence of plasticization phenomena. The permeability coefficient of N_2_, CH_4_ and H_2_ increased with increasing temperature while CO_2_ permeability decreased with increasing temperature as expected for a glassy polymer. The diffusion and solubility coefficients were also analysed individually and compared with other polymers of intrinsic microporosity. From these results, the activation energies of permeation, diffusion and sorption enthalpies were calculated using an Arrhenius equation.

## 1. Introduction

Membranes are one of the most promising technologies to compete with conventional separation processes for gas separations including post- and pre-combustion carbon capture. Studies on the use of polymeric membranes in an Integrated Gasification Combined Cycle (IGCC) power plant [1,2,3] show their viability and their competitiveness with the currently more developed solvent-based technology. Process simulations [1] have shown an advantage for hydrogen selective materials for this application and new membrane materials are currently under development [3]. The performance of the materials in the relatively harsh conditions of the separation (50 bars and 200 °C) needs to be investigated before production can be scaled up [4].

**Figure 1:**
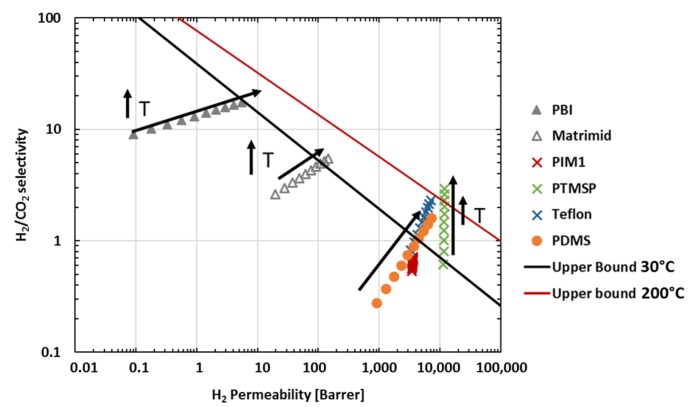
Influence of temperature on membrane performance (calculated from [7]) [6,8,9,10,11].

The increase of gas pressure can have a negative impact on membrane performance, due to plasticization effects. For glassy polymers, many gases, such as O_2_, N_2_ and H_2_, can permeate through the polymer without modifying the polymer’s properties due to their relatively low solubility in the polymer. Therefore, with the pressure’s increase, the gas permeability slightly decreases, as expected from the dual sorption—dual mobility model [5]. On the contrary, highly sorbing gases such as CO_2_ can induce a swelling of the polymer matrix, that is, plasticization, leading to a large increase of the gas permeability with increasing pressure. In addition, the influence of temperature on gas separation performance has been investigated for a large number of polymers. Depending on the polymer, the membrane performance can be improved by an increase of temperature as shown by the Robeson plot in Figure 1. Most polymers, including ultrahigh-free volume polymers such as PTMSP and Teflon AF, present higher hydrogen selectivity over CO_2_ at high temperature. For example, Merkel et al. [6] reported H_2_S, CO_2_, H_2_, N_2_ and CO permeation as a function of temperature up to 240 °C. At room temperature, PTMSP appears to be more permeable to the more condensable gases, such as CO_2_ and H_2_S than to H_2_. However, it becomes hydrogen selective at elevated temperatures.

For the first Polymer of Intrinsic Microporosity, PIM-1, Budd et al. [8] showed that the CO_2_ permeability coefficient decreased gradually as the temperature increased, whereas the H_2_ permeability coefficient increased. Thus, PIM-1 also becomes slightly more H_2_ selective at higher temperature. Recently, Fuoco et al. [12] studied the temperature dependence of gas permeation in triptycene-based ultrapermeable PIMs, such as PIM-TMN-Trip. With increasing temperature, the permeability coefficient increased for the bulkier penetrants (N_2_ and CH_4_), while for the faster penetrants (CO_2_ and O_2_) it decreased and for the very small penetrants (H_2_ and He) it was constant. Therefore, PIM-TMN-Trip became more selective to H_2_ at high temperature; these ultrapermeable polymers behave as microporous solids, in which the pore dimensions are rather large in comparison with the diffusing gas molecules. Such studies of the temperature and pressure dependence of transport properties are essential for understanding the behaviour of membranes over a wide range of conditions, in order to assist any consideration of industrial use.

**Figure 2:**
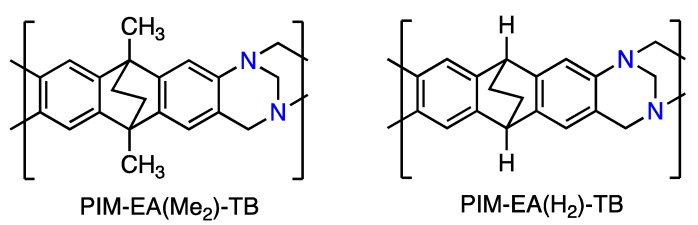
The chemical structure of PIM-EA(Me_2_)-TB and PIM-EA(H_2_)-TB.

Recently, a new type of PIM has been developed using a polymerization reaction based on the formation of the bridged bicyclic diamine called Tröger’s base (TB: 6H,12H-5,11-methanodibenzo[b,f][1,5]diazocine), such as PIM-EA(Me_2_)-TB [13] or PIM-EA(H_2_)-TB [14,15] (Figure 2). PIM-EA(Me_2_)-TB demonstrates at ambient temperature very fast gas permeability and good selectivity, surpassing the Robeson’s upper bound in the case of O_2_/N_2_, H_2_/N_2_ and H_2_/CH_4_ gas pairs [16,17]. This is due to the large diffusivity selectivity that favours transport of gas molecules of smaller kinetic diameters (H_2_, CO_2_) over that of larger molecules (N_2_, CH_4_).

PIM-EA(H_2_)-TB differs from PIM-EA(Me_2_)-TB only by the absence of methyl groups at the bridgehead (9,10) position of the ethanoanthracene (EA) unit, which modifies its chain packing in the solid state. PIM-EA(H_2_)-TB presents an inter-chain distance, d-space, of 7.7 Å and 32% free volume, whereas PIM-EA(Me)-TB has values of 11 Å and 30%, respectively [18]. With these differences, a higher separation performance for PIM-EA(H_2_)-TB is expected. However, few papers have been published on this polymer. Bernardo et al. [15] developed thin film composite based on PIM-EA(H_2_)-TB and they studied the impact of the residual casting solvent on the separation performance at 25 °C and 1 bar. In addition, Benito et al. [19] studied composite membranes based on a ultrathin layer of PIM-EA(H_2_)-TB for CO_2_/N_2_ separation at 35 °C and 3 bar.

Here we report a novel study on the permeation properties of PIM-EA(H_2_)-TB over a large temperature and pressure range for a series of gases (CO_2_, H_2_, N_2_ and CH_4_).

## 2. Experimental Section

**Figure 3:**
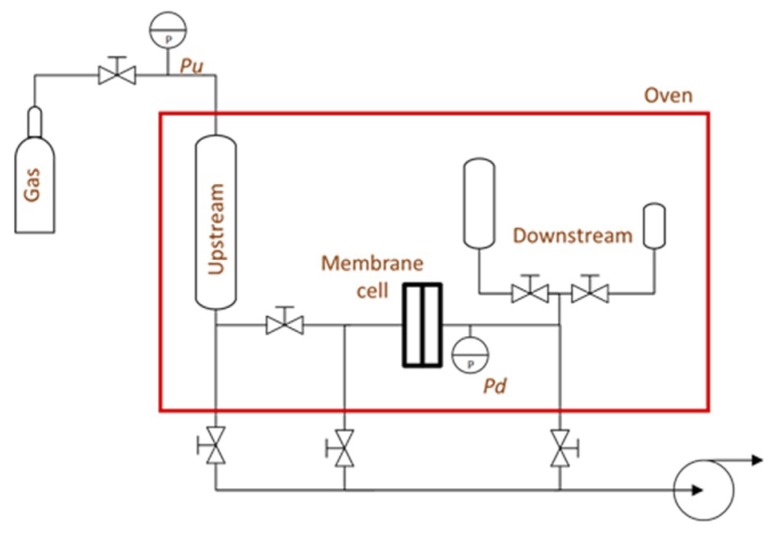
Constant volume-variable pressure apparatus.

The detailed synthetic procedure for making PIM-EA(H_2_)-TB and its structural characterization are reported elsewhere [15]. Robust flat films of thickness between 130 and 200 μm were cast from chloroform with their thickness determined using a digital micrometre (Mitutoyo, Kawasaki, Japan). The permeation properties were measured in a constant volume-variable pressure apparatus (Figure 3) using pure CO_2_, N_2_, CH_4_ and H_2_ (Table 1) with pressures up to 20 bar (10 bar for H_2_) and temperatures up to 200 °C.

For each measurement campaign (i.e., one gas and either variable T or variable P), the sample was carefully treated with methanol prior to the measurement in order to start from the same ageing history. The methanol treatment consists of soaking the sample in methanol for 2 h, drying it under ambient conditions for 20 min and under vacuum at 30 °C overnight. At the end of the campaign, the gas permeability at 30 °C and 1 bar was re-measured in order to check the absence of physical/chemical ageing. Moreover, each campaign’s duration was short, carried out over a maximum of 3 days in order to limit physical ageing. By using this procedure, the physical ageing was minimised and had no apparent impact on the results for permeability and selectivity.

The permeability was obtained from the evolution of pressure of the downstream side (MKS Baratron 615A (Andover, MA, USA)). The permeability coefficient, *P*, was determined from the slope of the pressure vs. time curve under steady state condition. Before each experiment, the apparatus was vacuum-degassed and a leak rate determined from the pressure increase in the downstream part. Three different downstream volumes could be selected accordingly to the permeation rate of the gas. 

The time lag, *θ*, was used to determine the diffusivity coefficient *D* (Equation (1)).

(1) $$D=\frac{l^{2}}{6\theta }$$

The solubility coefficient, *S*, for the gas in the polymer was evaluated indirectly, assuming the validity of the diffusion-solution mechanism (Equation (2)):

(2) $$S=\frac{P}{D}$$

The ideal selectivity between two gas species *i* and *j* is the ratio of the two single gas permeabilities (Equation (3)).

(3) $$\alpha_{ij}=\frac{P\left(i\right)}{P\left(j\right)}$$

## 3. Results

### 3.1. Permeability

Permeation measurements on methanol treated films of PIM-EA(H_2_)-TB were carried out using pure N_2_, H_2_, CO_2_ and CH_4_ at several pressures (1 to 20 bar) and temperatures (30 °C to 200 °C).

Table 2 reports the results from the time lag experiment at 30 °C and 1 bar.

PIM-EA(H_2_)-TB presents high CO_2_ and H_2_ permeability coefficients and good ideal selectivity over N_2_ and CH_4_. The order of gas permeabilities for PIM-EA(H_2_)-TB is CO_2_ > H_2_ > CH_4_ > N_2_, the same as that for PIM-1. CO_2_, which is the most condensable gas, is the most permeable due to the predominant role of solubility in PIMs [8]. In comparison with PIM-EA(Me_2_)-TB, the permeability coefficients obtained for PIM-EA(H_2_)-TB are lower. This can be explained by the methyl groups increasing the distance between polymer chains of PIM-EA(Me_2_)-TB, relative to PIM-EA(H_2_)-TB, which ensures higher free volume and, hence, higher permeability [16] but reduces selectivity.

**Figure 4:**
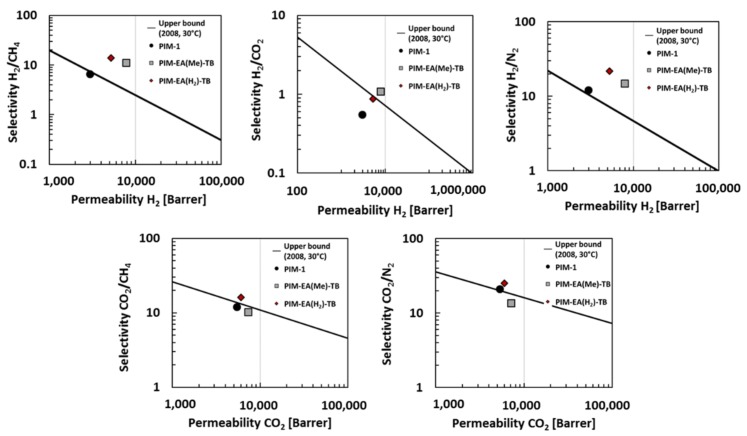
Robeson plots for H_2_/CH_4_, H_2_/N_2_, H_2_/CO_2_, CO_2_/CH_4_ and CO_2_/N_2_ for PIM-1 [8] (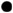), PIM-EA(Me_2_)-TB [16] (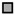) and PIM-EA(H_2_)-TB [our study] (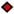) at 30 °C and 1 bar. The lines represents the 2008 upper bound for each gas pair [21].

Figure 4 shows the Robeson plots for five gas pairs, H_2_/CH_4_, H_2_/N_2_, H_2_/CO_2_, CO_2_/CH_4_ and CO_2_/N_2_.

As shown on Figure 4, the data for PIM-EA(H_2_)-TB are located above the 2008 upper bound for all five gas pairs. For H_2_/CH_4_ and H_2_/N_2_, they are clearly higher than for PIM-1 and PIM-EA(Me)-TB. This demonstrates the potential of PIM-EA(H_2_)-TB for industrial applications, such as carbon capture (CO_2_/N_2_ mixture), natural gas sweetening and biogas treatment (CO_2_/CH_4_ mixture) or hydrogen recovery (H_2_/CH_4_ mixture).

### 3.2. Diffusivity and Solubility Coefficients

The gas transport in PIM-EA(H_2_)-TB was analysed using the solution-diffusion model (Equation (2)), to provide the diffusivity and sorption coefficients (Table 3).

The diffusivity and solubility values of PIM-EA(H_2_)-TB are similar to those of polymers from the same family (PIM-EA(Me)-TB) [16] with a very high value of CO_2_ solubility coefficient. This affinity towards CO_2_ may be enhanced by the presence of the amine groups in the TB moiety.

**Figure 5:**
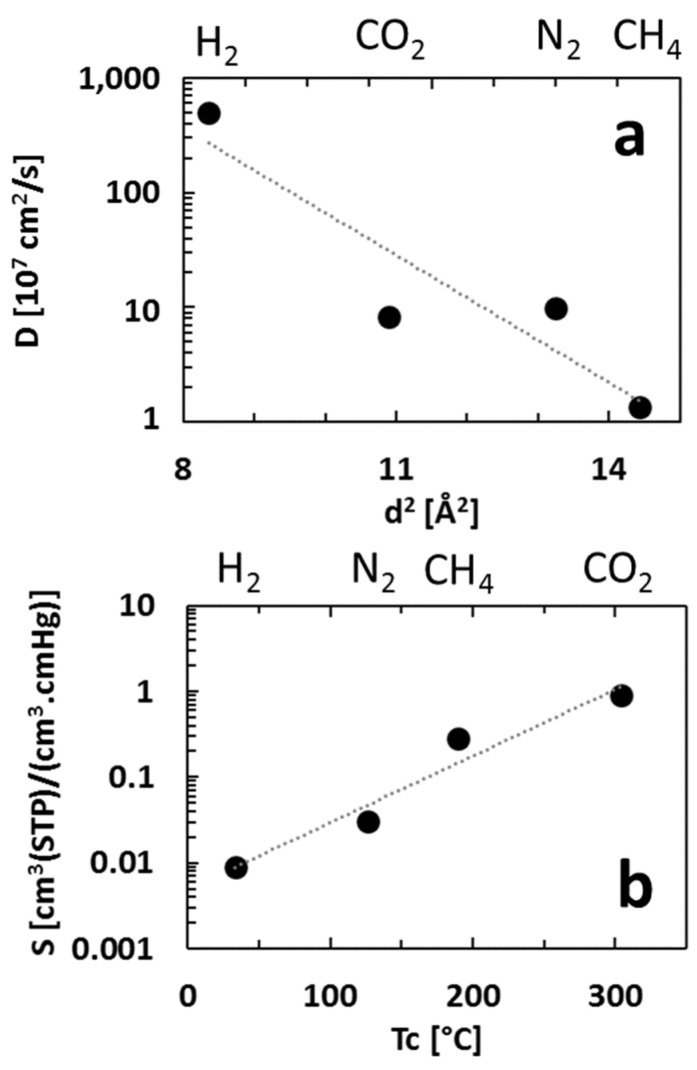
Diffusivity (**a**) and solubility (**b**) coefficients of PIM-EA(H_2_)-TB for H_2_, CO_2_, CH_4_ and N_2_ at 30 °C and 1 bar.

Diffusivity and solubility data are plotted in Figure 5 as correlations of log *D* versus *d*^2^ and log *S* versus *T_c_*, respectively, where *d* is the kinetic diameter and *T_c_* is the critical temperature of the gases.

Figure 5a shows that the diffusivity coefficient of PIM-EA(H_2_)-TB decreases with increasing molecular size of the permeate. Larger molecules interact with more segments of the polymer chains than do smaller molecules and thus the mobility selectivity always favours the passage of smaller molecules over larger ones [20]. Moreover, this decrease is large due to the glassy state of the polymer where the highly rigid polymer chains of PIM-EA(H_2_)-TB are essentially fixed and do not move readily to accommodate the transport of larger molecules. It is noteworthy that the value of diffusivity for CO_2_ is slightly lower than for N_2_. Generally, in polymers, the smaller molecule, that is, CO_2_, is expected to diffuse faster than N_2_, which is a larger molecule. This unusual inversion is found for polymer with high CO_2_ affinity [13,17,22] and is caused by the specific interaction between CO_2_ and amine groups slowing diffusion [23].

The sorption coefficient of the gas within PIM-EA(H_2_)-TB increases with its critical temperature (i.e., its condensability) as is usually observed for polymers (Figure 5b).

### 3.3. Effect of Pressure

**Figure 6:**
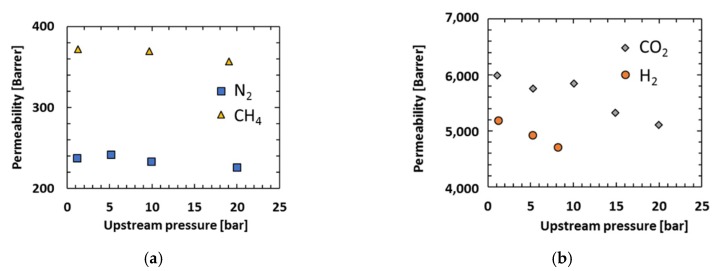
Permeability coefficients of PIM-EA(H_2_)-TB for CH_4_, N_2_(**a**) and H_2_, CO_2_ (**b**) at 30 °C.

The permeability coefficients of each gas were measured as a function of upstream feed pressure. The measurements were carried out with H_2_, CO_2_, CH_4_ and N_2_ at 30 °C and pressures up to 20 bar (10 bar for H_2_) (Figure 6).

The permeability of nitrogen is constant with increasing pressure while CO_2_ and CH_4_ permeabilities decrease with increasing pressure, which is classical behaviour for glassy polymers [24] and is due to the filling of Langmuir sorption sites. At higher pressures, the contribution of the Langmuir region to the overall permeability is weaker and gas permeability approaches a constant value associated with simple dissolution (Henry’s law) transport. In contrast to the majority of glassy polymers, PIM-EA(H_2_)-TB does not exhibit the typical increase in CO_2_ permeability associated with “plasticization” in the high pressure range for CO_2_. A similar behaviour has been also noted for other polymers of intrinsic microporosity, such as PIM-1 or PIM-EA(Me)-TB [17,24,25]. However, the decrease in H_2_ permeability is higher than expected [25].

Despite the decrease of permeability coefficients, the ideal selectivities of PIM-EA(H_2_)-TB stay constant with the increase of the feed pressure (Table 4). However, it should be noted that ideal selectivity is usually not representative of behaviour at high pressure in mixed gas systems due to the interactions between different gases.

### 3.4. Effect of Temperature

**Figure 7:**
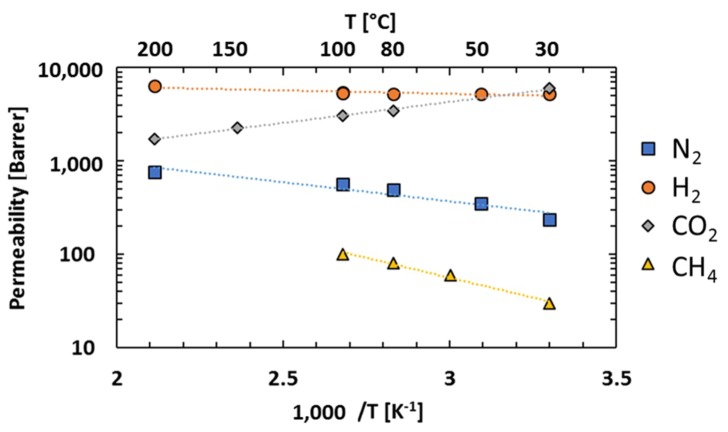
Permeability coefficients of N_2_, CO_2_, H_2_ and CH_4_ as a function of the inverse absolute temperature (at 1 bar) (The dotted lines represent the best curve-fits of the experimental data with Arrhenius equation).

The temperature effect on gas permeability through PIM-EA(H_2_)-TB was studied over a temperature range of 30–200 °C for pure gas at different pressures. The values of the permeability coefficients are summarised in the Table S1. Figure 7 shows the permeability coefficient of N_2_, CO_2_, H_2_ and CH_4_ as a function of the inverse absolute temperature at 1 bar.

The permeability coefficient of N_2_, CH_4_ and H_2_ increases with increasing temperature while for CO_2_ it decreases with increasing temperature. In order to explore the temperature dependence of the gas permeability, the data were correlated with the Arrhenius equation.

(4) $$P=P_{0}exp\left(-\frac{E_{p}}{RT}\right)$$

where *P*_0_ is the pre-exponential factor ((cm^3^(STP)·cm)/(cm^2^·s·cmHg)), *E_p_* is the activation energy of permeation (J/mol), *T* is the temperature (K) and *R* is the ideal gas constant (8.314 kJ/(mol·K)). *E_p_* for the transport of each gas through PIM-EA(H_2_)-TB were determined from the slopes (−*E_P_*/*R*) of the best curve-fits through the permeation data in Figure 7. The *E_p_* values at 1 bar are summarized in Table 5.

PIM-EA(H_2_)-TB presents high values for the activation energy of permeation for N_2_ and CH_4_, which means that the permeability coefficients depend strongly on the temperature. On the contrary, for the smaller gases, such as H_2_, *E_P_* is close to zero as the dependence on temperature is much weaker. For CO_2_, the activation energy of permeation is negative. This behaviour is routinely observed for microporous solids, such as PIM-1, PIM-TMN-Trip and PTMSP, in which the pore dimensions are relatively large in comparison with the diffusing gas molecules [11].

Since the gas transport in a microporous membrane is based on a solution-diffusion mechanism, the impact of temperature on the permeation can be better understood when looking at diffusion and solubility individually. The activation energy of permeation can be represented as the sum of the activation energies of diffusion, *E_D_* and sorption Δ*H_s_*. Table 6 lists the activation energies of gas permeation and diffusion as well as the enthalpy of sorption of all the gases in PIM-EA(H_2_)-TB. For all the gases at 1 bar, the activation energy of diffusion, *E_D_*, is positive, which means that the diffusivity increases with the temperature, which is expected as the main effect of increasing the temperature is an increase of molecular vibrations which facilitates diffusion. In contrary, the sorption enthalpy, Δ*H_s_*, is negative as expected since the sorption is an exothermic process.

For CH_4_, N_2_ and H_2_, the absolute value of *E_D_* is greater than Δ*H_s_* and so the energy of activation *E_p_* is positive, which means that diffusion rather than sorption dominates the response of permeation to temperature. For CO_2_, the absolute value of *E_D_* is smaller than Δ*H_s_*, which induces a negative activation energy *E_P_*. The CO_2_ transport is mainly influenced by the gas solubility, which is characteristic of microporous polymer, with similar results being found for PIM-1 and PTMSP [5,8,11].

**Figure 8:**
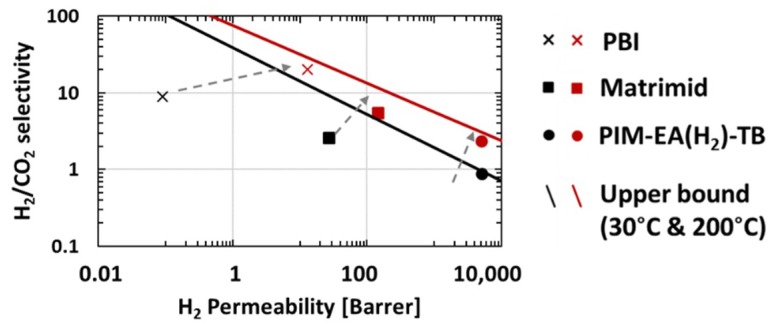
H_2_/CO_2_ separation performances of PBI (Cross), Matrimid (Square) and PIM-EA(H_2_)-TB (circle) at 1 bar/30 °C (Black dot) and at 10 bar/200 °C (Red dot). Upper bound at 200 °C recalculated from [7].

**Figure 9:**
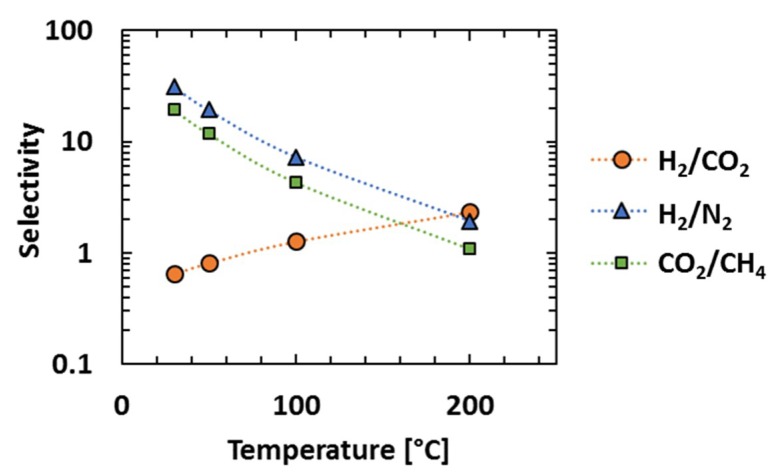
Selectivity of PIM-EA(H_2_)-TB as the function of temperature at 10 bar.

Based upon these effects, the increase of temperature improves H_2_/CO_2_ selectivity modestly moving the data for PIM-EA(H_2_)-TB close to the 200 °C upper bound (Figure 8, however, even its enhanced high temperature selectivity (~2) is insufficient for viable pre-combustion application. In contrast, the selectivity for CO_2_ or H_2_ over N_2_ or CH_4_ decreases dramatically at higher temperatures suggesting that optimal performance is obtained at lower temperatures (Figure 9).

## 4. Conclusions

Transport properties of permeability, diffusivity and solubility of PIM-EA(H_2_)-TB have been determined for H_2_, N_2_, CH_4_ and CO_2_ over a range of pressures and temperatures. This PIM presents high CO_2_ and H_2_ permeability coefficients, which allows it to have good ideal selectivity over N_2_. PIM-EA(H_2_)-TB exhibits the classical behaviour of a glassy polymer, with the decrease of diffusivity coefficient with increasing penetrant molecular size and the increase of sorption coefficient gas with increasing condensability of the permeant. However, no increase in CO_2_ permeability due to plasticization is noted over the range of pressure tested. The permeability coefficient of N_2_, CH_4_ and H_2_ increase with increasing temperature while for CO_2_ the permeability decreases with increasing temperature, which is classically observed for microporous materials. Therefore, the separation performance of PIM-EA(H_2_)-TB for H_2_/CO_2_ is reversed at high temperature and maintained also at high pressure. This suggests that, after further development to enhance absolute selectivity of H_2_ over CO_2_, PIMs could become good candidates for membrane materials for use in pre-combustion CO_2_ capture. For other gas separations, better performance is obtained at lower temperatures.

## Figures and Tables

**Table 1:** Kinetic diameter and critical temperature [20].

Gas	Kinetic Diameter (*d*) (Å)	Critical Temperature (*T_c_*) (K)
H_2_	2.89	33.2
N_2_	3.64	126.2
CH_4_	3.8	190.6
CO_2_	3.3	304.2

**Table 2:** Gas permeabilities and ideal selectivities (CO_2_/Gas, H_2_/Gas) for MeOH treated film PIM-EA(H_2_)-TB at 30 °C, 1 bar (Errors calculated by statistical analysis of repeated measurements from separately prepared films (between 3 and 5)).

30 °C, 1 bar		N_2_	H_2_	CO_2_	CH_4_
PIM-1 [8]	Permeability (Barrer)	252	2936	5303	440
	Selectivity CO_2_/Gas	21	1.8	-	12
	Selectivity H_2_/Gas	12	-	0.5	6.7
PIM-EA(Me_2_)-TB [16]	Permeability (Barrer)	525	7760	7140	699
	Selectivity CO_2_/Gas	13.6	0.9	-	10
	Selectivity H_2_/Gas	14.8	-	1.1	11
PIM-EA(H_2_)-TB (This study)	Permeability (Barrer) (± Error)	238 (± 3%)	5188 (± 1%)	5990 (± 1%)	372 (± 3%)
	Selectivity CO_2_/Gas	25	1	-	16
	Selectivity H_2_/Gas	22	-	1	14

**Table 3:** Diffusivity and solubility coefficients for MeOH treated film PIM-EA(H_2_)-TB, at 30 °C, 1 bar (Errors calculated by statistical analysis of repeated measurements from separately prepared films).

30 °C, 1 bar	N_2_	H_2_	CO_2_	CH_4_
*D* (10^−7^ cm^2^/s) (± Error)	9.7 (± 12%)	500.0 (± 9%)	8.2 (± 3%)	1.3 (± 11%)
*S* (cm^3^(STP)/(cm^3^·cmHg)) (± Error)	3 × 10^−2^ (± 15%)	9 × 10^−3^ (± 10%)	9 × 10^−1^ (± 4%)	3 × 10^−1^ (± 14%)

**Table 4:** Selectivity of PIM-EA(H_2_)-TB for CH_4_, N_2_, H_2_, CO_2_ at 30 °C for different pressures.

Selectivity, 30 °C	H_2_/CO_2_	H_2_/N_2_	H_2_/CH_4_	CO_2_/N_2_	CO_2_/CH_4_	CH_4_/N_2_
1 bar	1	22	14	25	16	2
5 bar	1	20	-	24	-	-
10 bar	1	20	14	25	16	2
20 bar	-	-	-	23	14	2

**Table 5:** Activation energy of gas permeation for PIM-EA(H_2_)-TB, PIM-1, PIM-TMN-Trip and PTMSP.

Gas	*E_P_* (kJ/mol)			
	PIM-EA(H_2_)-TB (This Study)	PIM-1 [26]	PIM-TMN-Trip [12]	PTMSP [26]
H_2_	0.5	−0.4	−2.8	−2.1
N_2_	8.6	14.3	4.4	−3.5
CH_4_	13.1	19.4	9.5	−5.3
CO_2_	−8.6	−1	−7.7	−11.7

**Table 6:** Activation energies for gas permeation (*E_p_*), for diffusion (*E_d_*) and for sorption (Δ*H_s_*) of PIM-EA(H_2_)-TB for N_2_, CO_2_, H_2_ and CH_4_ at 1 bar.

1 bar	*E_P_* (kJ/mol)	*E_D_* (kJ/mol)	Δ*H_s_* (kJ/mol)
CO_2_	−8.6	8.1	−16.7
N_2_	8.6	18.5	−9.9
H_2_	0.5	5.2	−4.6
CH_4_	13.1	17.9	−4.8

## Supplementary Materials

The following are available online at http://www.mdpi.com/2077-0375/8/4/132/s1, Table S1: Gas permeability coefficients of N_2_, CO_2_, H_2_ and CH_4_ for temperatures between 30 °C and 200 °C and pressure between 1 bar and 20 bar.
