# Supplementary material for: Temperature and Pressure Dependence of Gas Permeation in a Microporous Tröger’s Base Polymer
Source: Membranes (Basel). 2018 Dec 14;8(4):132. doi: 10.3390/membranes8040132 (PMC6316465; doi:10.3390/membranes8040132)
Supplement: Supplementary file 1 [file membranes-08-00132-s001.pdf]

membranes

Supplementary Materials: Temperature and Pressure Dependence of Gas Permeation in a Microporous Tröger's Base Polymer

Elsa Lasseuguette, Richard Malpass-Evans, Mariolino Carta, Neil B. McKeown and Maria-Chiara Ferrari

Table S1. Gas permeability coefficients of N_2, CO_2, H_2 and CH_4 for temperatures between 30 °C and 200 °C and pressure between 1 bar and 20 bar.

CO_2	Perm (Barrer)			
	30°C	50°C	100°C	200°C
1 bar	5862	4745	3088	1717
10 bar	7305	5909	3841	2132
20 bar	4819	3959	2656	1540
N_2	Perm (Barrer)			
	30°C	50°C	100°C	200°C
1 bar	268	331	508	913
10 bar	234	305	525	1101
20 bar	226	296	510	1073
H_2	Perm (Barrer)			
	30°C	50°C	100°C	200°C
1 bar	5158	5229	5377	5587
10 bar	4714	4761	4859	4995
20 bar	-	-	-	-
CH_4	Perm (Barrer)			
	30°C	50°C	100°C	200°C
1 bar	380	525	1012	2481
10 bar	375	499	891	1968
20 bar	363	488	890	2023
